# Autophagy is activated to protect against endotoxic acute kidney injury

**DOI:** 10.1038/srep22171

**Published:** 2016-02-26

**Authors:** Shuqin Mei, Man Livingston, Jielu Hao, Lin li, Changlin Mei, Zheng Dong

**Affiliations:** 1 Kidney Institute, Department of Nephrology, Shanghai Changzheng Hospital, Second Military Medical University, Shanghai, China; 2 Department of Cellular Biology and Anatomy, Medical College of Georgia, Georgia Regents University and Charlie Norwood VA Medical Center, Augusta, Georgia; 3 Institute of Nephrology, The Second Xiangya Hospital, Central South University, Changsha, China

## Abstract

Endotoxemia in sepsis, characterized by systemic inflammation, is a major cause of acute kidney injury (AKI) in hospitalized patients, especially in intensive care unit; however the underlying pathogenesis is poorly understood. Autophagy is a conserved, cellular catabolic pathway that plays crucial roles in cellular homeostasis including the maintenance of cellular function and viability. The regulation and role of autophagy in septic or endotoxic AKI remains unclear. Here we show that autophagy was induced in kidney tubular cells in mice by the endotoxin lipopolysaccharide (LPS). Pharmacological inhibition of autophagy with chloroquine enhanced LPS-induced AKI. Moreover, specific ablation of autophagy gene 7 (Atg7) from kidney proximal tubules worsened LPS-induced AKI. Together, the results demonstrate convincing evidence of autophagy activation in endotoxic kidney injury and support a renoprotective role of autophagy in kidney tubules.

Sepsis with endotoxemia was firstly identified in the 19^th^ century as a severe syndrome characterized by overwhelming whole body inflammation followed by immunosuppression. Based on the criteria established by the American College of Chest Physicians/Society of Critical Care Medicine Consensus conference in 1991, sepsis, severe sepsis and septic shock were presented to better elaborate the process and progression of “sepsis”^1^. The incidence of sepsis has been increasing rapidly during the past years, despite more clinically progressive therapies and treatments are applied. Sepsis causes the damage of multiple organs that is frequently associated with patient death. Acute kidney injury (AKI) is one of the well-documented complications in sepsis that is associated with high morbidity and mortality in hospitalized patients, especially in intensive care unit (ICU)^2^,^3^. A significant body of evidence shows that septic AKI involves a complex pathogenesis, including inflammation, tubular epithelial cell injury, and endothelial and vascular dysfunction^4^,^5^,^6^. There is an urgent need for effective therapeutics of septic or endotoxic AKI^7^.

Autophagy is a tightly regulated, cellular process of catabolism that is conserved from yeast to mammals and plays crucial roles in cellular homeostasis via the degradation of cytoplasmic components^8^,^9^,^10^. Autophagy related genes (Atgs) are the core components of the molecular machinery of autophagy that are responsible for the formation of autophagic vesicles of various stages from isolation membrane, autophagosomes, to autolysosome^8^,^9^,^10^. Although initially described as a response to starvation, autophagy is now known to be a general cellular response to stress. As such, autophagy has been implicated in a variety of physiological as well as disease conditions, such as cancers, infectious diseases, cardiovascular dysfunctions, and neurodegenerative disorders^11^.

In ischemic and nephrotoxic models of AKI, we and others have demonstrated the activation of autophagy in kidney tissues and cells^12^,^13^,^14^,^15^,^16^. Notably, by using pharmacological inhibitors and autophagy-deficient models further demonstrated a protective role of autophagy in these AKI models^12^,^13^,^14^,^15^,^16^. However, it remains much less clear about autophagy in septic or endotoxic AKI. Is autophagy activated? If yes, when and in what cells? And what role does autophagy play in the pathogenesis of septic or endotoxic AKI? In order to answer these questions, we tested the effect of lipopolysaccharide (LPS), a major endotoxin in sepsis from the cell wall of Gram-negative bacteria.

## Results

### Effects of LPS on renal function and general health index

**Figure 1:**
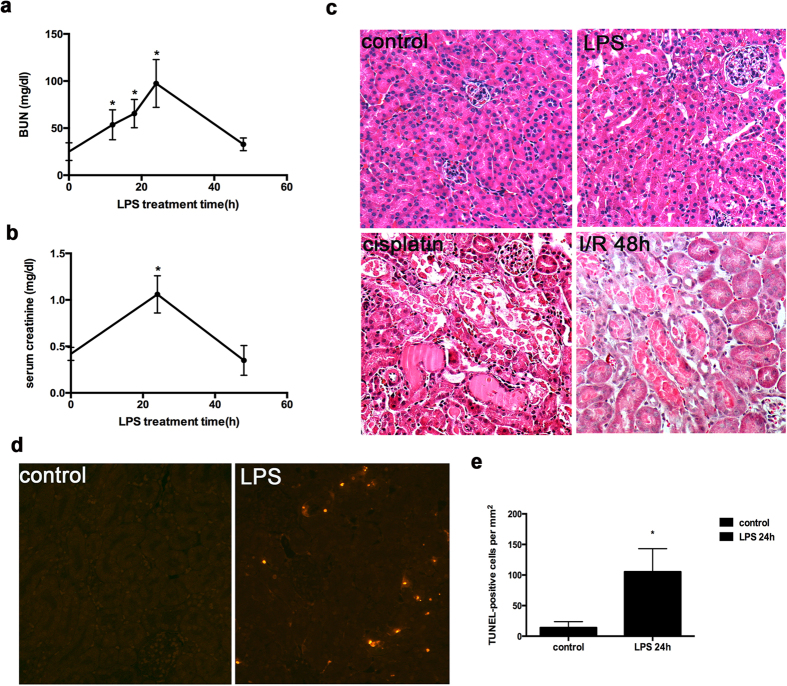
LPS-induced AKI in mice. C57BL/6 mice (male, 8–12 weeks old) were divided into 4 groups and subjected to the following treatment: 1) control (injected with saline); 2) LPS treatment for 4 hours; 3) LPS treatment for 24 hours; 4) LPS treatment for 48 hours. (**a**,**b**) Blood samples were collected for BUN and creatinine measurement. *P < 0.05 significant difference vs. control. (**c**) H&E staining of renal histology of AKI following LPS (10 mg/kg, 24 hours), cisplatin (25 mg/kg, 4 days), and 28 minutes of bilateral renal ischemia followed by 48 hours reperfusion (×200). (**d**) Representative images of TUNEL staining of kidney tissues from control and LPS (24 hours)-treated mice. (**e)** TUNEL positive cells were counted and expressed as mean ± SD per square mm area. *P < 0.05 significant difference vs. control.

Following LPS injection, mice appeared inactive at 8 h and severely ill at 24 h. They developed low temperature, diarrhea, increased eye secretions and lethargic, which were corresponding to the decline of renal function. Their plasma BUN and serum creatinine increased respectively from 25.07 ± 9.38 mg/dl and 0.42 ± 0.07 mg/dl at 0 h to 97.49 ± 25.45 mg/dl and 1.06 ± 0.2 mg/dl at 24 h. Thereafter, BUN and Serum creatinine decreased and by 48 h, BUN and serum creatinine were 32.86 ± 6.78 mg/dl and 0.35 ± 0.16 mg/dl, respectively (Fig. 1a,b). LPS also induced weight loss compared to control group (data not shown).

### Comparison of AKI induced by LPS, cisplatin and ischemia-reperfusion

Cisplatin nephrotoxicity and ischemia/reperfusion (I/R) induced massive tubule disruption, dilation, lysis, and cast formation in kidneys (Fig. 1c)^17^,^18^, while LPS just induced a moderate tubule injury. The injury induced by LPS was shown mainly in renal cortex and the outer stripe of outer medulla and was characterized by focal tubular cell swelling, dilatation, vacuolization, and occasional detachment (Fig. 1c). Based on the evaluation of the blinded observer, the injury was detected in around 15% renal tubules after 24 h of LPS treatment, which was much less than cisplatin and I/R induced AKI. By TUNEL assay, we also detected apoptosis in kidney tissues of LPS-treated mice (Fig. 1d,e).

### Autophagy is induced in LPS-induced AKI

**Figure 2:**
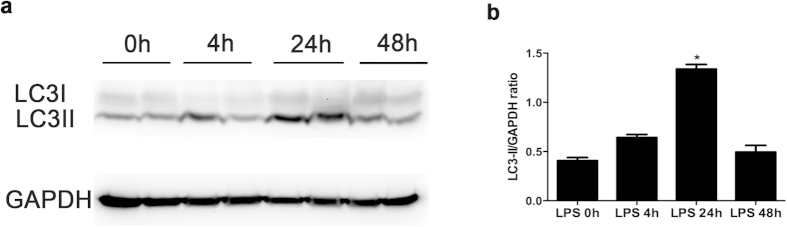
LC3II accumulation in kidney tissues during LPS treatment. **(a**) Representative immunoblots. (**b**) The LC3II and GAPDH signals in immunblots were analyzed by densitometry to calculate the ratio to expressed as mean ± SD. *P < 0.05, significantly different from other groups.

A biochemical hallmark of autophagy is the formation of LC3 II that is converted from LC3 I via cleavage and lipidation and incorporated into autophagosomes^19^. We therefore initially examined autophagy in kidney tissues by immunoblot analysis of LC3. As shown in Fig. 2a, the accumulation of LC3 II was slightly increased after LPS treatment of 4 h, reached to the peak at 24 h, and then gradually reduced to nearly the control level. The results were confirmed by evaluating the LC3II/GAPDH ratio of immunoblots (Fig. 2b).

**Figure 3:**
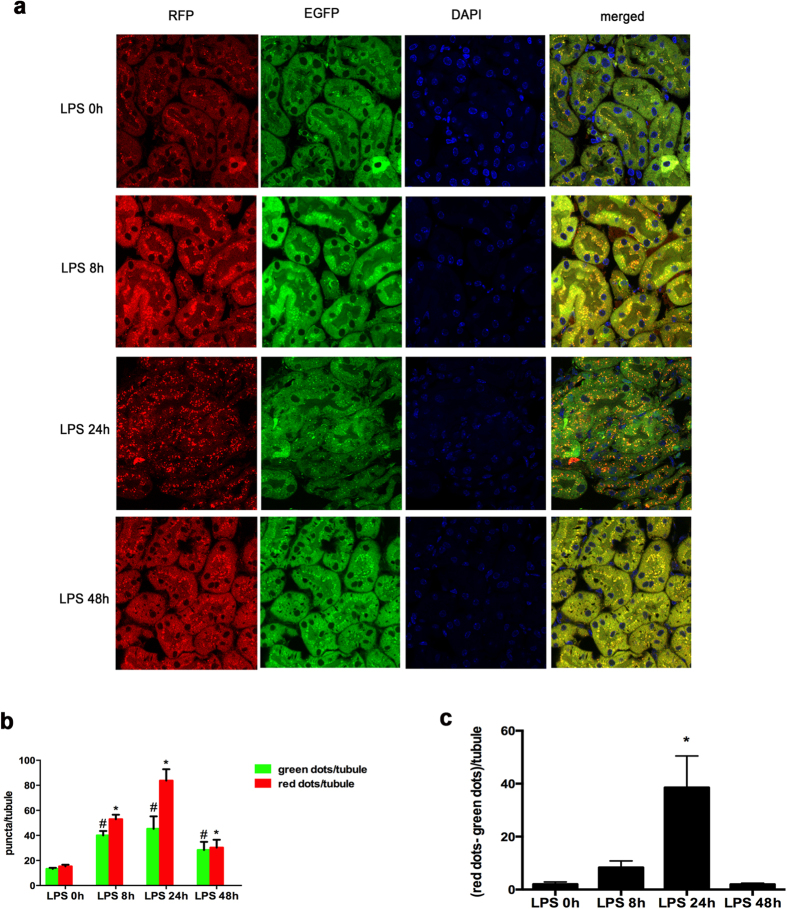
Dynamics of autophagy during LPS-induced AKI shown by CAG-RFP-EGFP-LC3 mice. **(a**) Representative images to show the dynamic changes of RFP and EGFP-LC3 puncta in kidney tubules during LPS treatment. (**b**) Quantification of EGFP and RFP puncta per tubule. Data were expressed as mean ± SD. *P < 0.05, significant difference in RFP-LC3 puncta vs. control group; ^#^*P* < 0.05, significant difference in EGFP-LC3 puncta vs. control. (**c**) The number of RFP-red puncta was subtracted by the number of EGFP-green puncta to estimate the number of lysosomes. Data were expressed as mean ± SD. *P < 0.05, significant difference vs. other groups.

In order to further verify autophagy induction and understand the dynamics of autophagy in LPS-induced AKI, we used autophagy reporter *CAG-RFP-EGFP-LC3* mice, which express a tandem RFP-GFP-LC3 fusion protein under the control of the CAG promoter^20^,^21^. The RFP-GFP-LC3 protein shows both RFP red fluorescence and GFP green fluorescence in neutral to basic pH, but GFP fluorescence is quenched when pH is low or acidic. Accordingly, autophagosomes would show both RFP and GFP fluorescence due to the neutral pH, but autolysosomes would only show red RFP signal due to their acidic pH of 4–5^20^,^21^,^22^. As shown in Fig. 3a, only a few RFP/GFP puncta were shown in control kidney tissues (LPS 0 h), while more RFP and EGFP puncta appeared after 8 h of LPS treatment and much more puncta per tubule appeared at 24 h of treatment. Furthermore, after 24 h of LPS treatment, a large percentage of puncta only showed red RFP signal, indicating the progress into autolysosome. Interestingly, at the 48 h the number of RFP and EGFP puncta decreased as compared with 24 h, suggesting the completion of autophagic degradation (Fig. 3a). Quantification of the puncta further verified the dynamic changes of autophagy, starting at the early time-point of 8 h, reaching a peak at 24 h, and then completing at 48 h (Fig. 3b). By subtracting the number of EGFP-green puncta from that of RFP-red puncta, we estimated the number of autolysosomes, which reached the highest level at 24 h of LPS treatment (Fig. 3c).

### Inhibition of autophagy by chloroquine aggravates LPS-induced AKI in C57BL/6 mice

**Figure 4:**
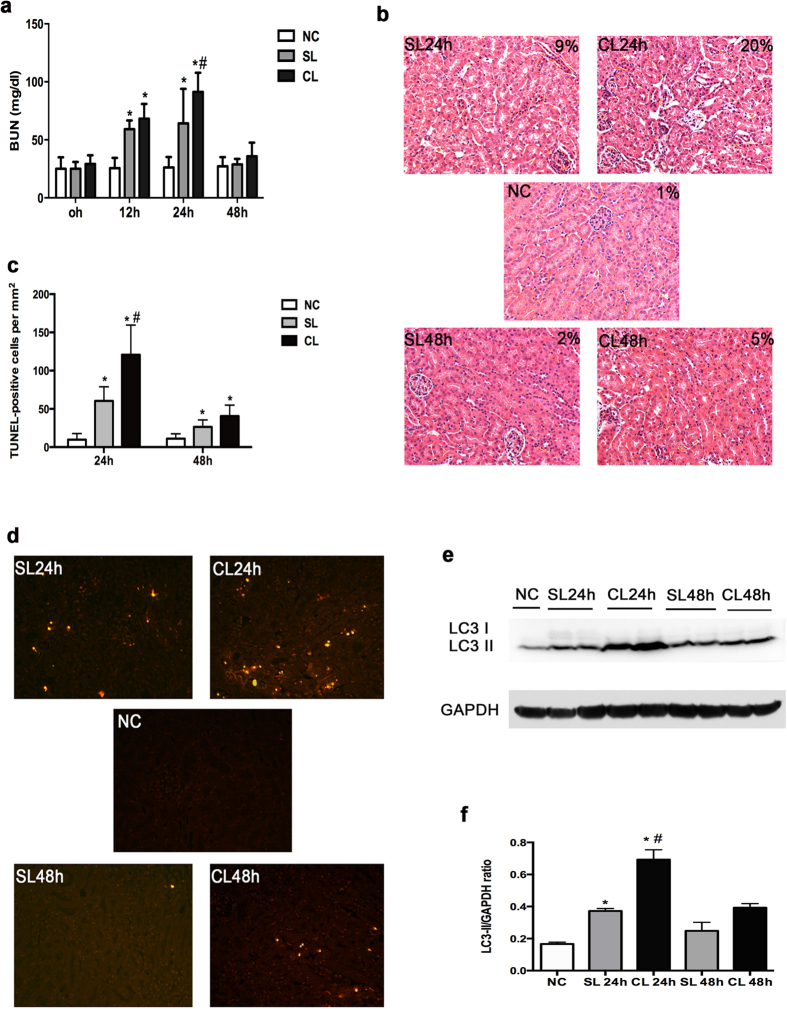
Chloroquine enhances LPS-induced AKI in C57BL/6 mice. C57BL/6 mice (male, 8–12 weeks old) were divided into 5 groups to subject to following treatments: 1) NC (normal control: injected with saline); 2) SL24 h (saline + LPS treatment for 24 hours); 3) CL24 h (chloroquine + LPS treatment for 24 hours); 4) SL48 h (saline + LPS treatment for 48 hours); 3) CL48 h (chloroquine + LPS treatment for 48 hours). (**a**) Blood samples were collected for BUN analysis. *P < 0.05, significant difference vs. NC group; ^#^*P* < 0.05, significant difference vs. SL. (**b**) Representative histology by H&E staining (×200). (**c**) Quantification of TUNEL positive cells in kidney tissues. Data were expressed as mean ± SD. *P < 0.05, significant difference vs. NC group; ^#^*P* < 0.05, significant difference vs. SL group. (**d**) Representative images of TUNEL staining. (**e**) Representative immunoblots of LC3 and GAPDH in kidney tissues. (**f**) The LC3II and GAPDH signals in immunoblots were analyzed by densitometry to calculate the ratio. Data were expressed as mean ± SD. *P < 0.05, significant difference vs. NC group; ^#^*P* < 0.05, significant difference vs. SL group.

Although we confirmed the activation of autophagy, the role of autophagy in LPS-induced AKI was still poorly understood. In order to address this question, we initially tested the effect of chloroquine, a pharmacological inhibitor of autophagy that increases the pH of lysosome to prevent autolysosome degradation. As shown in Fig. 4a, compared to control (NC), LPS treatment increased BUN after 12 h and 24 h (SL). Chloroquine further increased BUN during LPS treatment at these two time points. For example, BUN was 64.17 ± 29.78 mg/kg at 24 h of LPS treatment and further increased to 91.25 ± 16.51 mg/kg with chloroquine. At 48 h after LPS treatment, BUN returned to basal level regardless of chloroquine (Fig. 4a).

Consistent with the kidney function results, histological analysis of renal cortex and out medulla showed more severe injury in the chloroquine + LPS (CL) group, especially at 24 h. In the presence of chloroquine, more tubules lost brush border and some tubules showed cell lysis (Fig. 4b). By quantification, the percentage of injured tubules after LPS treatment was around 9% and 20% in LPS-only (SL) and LPS + chloroquine (CL) groups, respectively (Fig. 4c). By TUNEL staining, we also detected more apoptotic cells in LPS + chloroquine (CL) tissue than LPS-only (SL) tissue (Fig. 4d,c). Of note, chloroquine alone did not have significant toxicity or side effect in kidney as shown in our previous work^12^. To verify the inhibitory effect of chloroquine on renal autophagy, we analyzed the accumulation of LC3II. Because chloroquine inhibits autophagy at the autolysosome level, it is expected to increase LC3II accumulation. Indeed, chloroquine induced higher accumulation of LC3II during LPS treatment (Fig. 4e,f). Thus, chloroquine inhibited renal autophagy during LPS treatment and worsened LPS-induced AKI, suggesting that autophagy may play a renoprotective effect in this model.

### LPS-induced renal autophagy is inhibited in proximal tubule-specific Atg7- knockout mice

**Figure 5:**
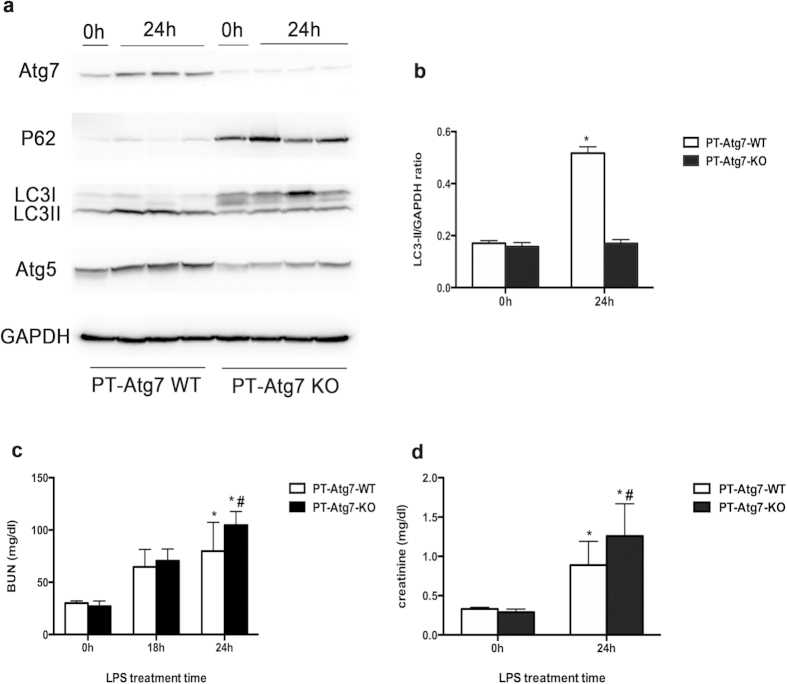
Suppression of autophagy and decrease of renal function in PT-Atg7-KO mice after LPS treatment. PT-Atg7-KO mice and wild-type PT- Atg7-WT littermates were treated with 10 mg/kg LPS. (**a**) Whole tissue lysate of the kidney was collected for immunoblot analysis of Atg7, p62, LC3, and GAPDH. (**b**) The LC3II and GAPDH signals in immunoblots were analyzed by densitometry to calculate the ratio to express as mean ± SD. *P < 0.05, significant difference between PT-Atg7-WT and PT-Atg7-KO group. (**c**,**d**) Blood samples were collected for BUN and creatinine measurement. *P < 0.05, significant difference between PT-Atg7-WT and PT-Atg7-KO group.

In order to further determine the effect of autophagy in LPS-induced AKI, we used proximal tubule-specific Atg7-knockout (PT-Atg7-KO) mice. As shown in Fig. 5a, kidney tissue of PT-Atg7-KO mice showed markedly less Atg7 expression as compared with the wild-type (PT-Atg7-WT) group. PT- Atg7-KO tissue also showed the accumulation of p62, a known autophagic substrate protein^23^. Consistently, PT-Atg7-KO kidney tissues also had much less LC3II accumulation following LPS treatment (Fig. 5a,b), further confirming that renal autophagy was suppressed in this model.

**Figure 6:**
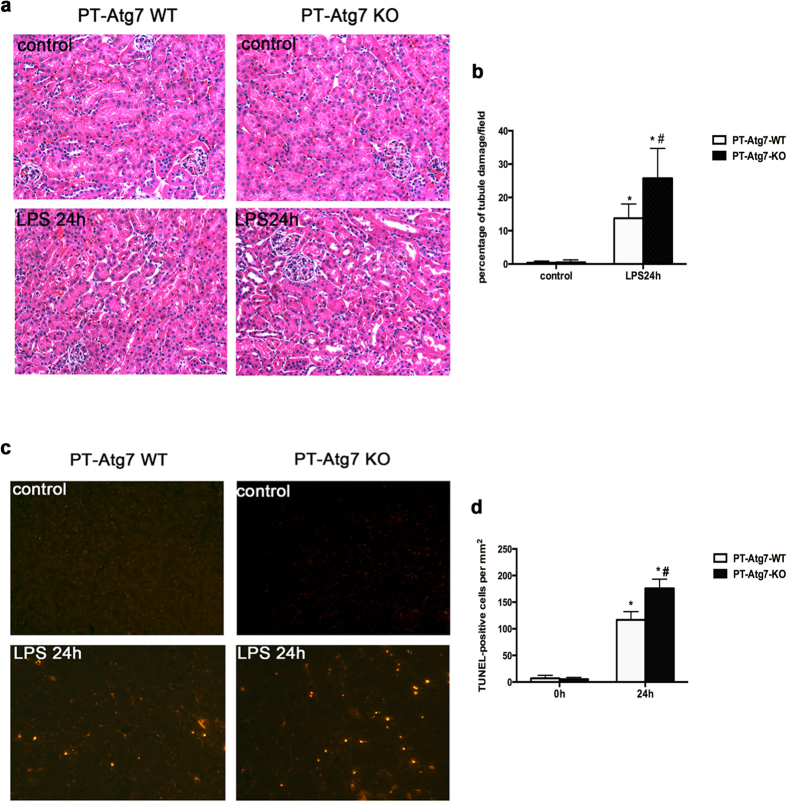
LPS-induced AKI is more severe in PT-Atg7-KO mice PT-Atg7- KO mice and wild-type PT-Atg7-WT littermates were injected to 10 mg/kg LPS. (**a**) Representative histology shown by H&E staining (×200). (**b**) Percentage of injured tubules evaluated by counting the renal tubules with signs of injury. Data were expressed as mean ± SD. *P < 0.05, significant difference vs. their corresponding control; ^#^*P* < 0.05, significant difference between PT-Atg7-WT and PT-Atg7-KO group. (**c**) Representative images of TUNEL staining. (**d**) Quantification of TUNEL positive cells in kidney tissues. Data were expressed as mean ± SD. *P < 0.05, significant difference between PT-Atg7-WT and PT-Atg7-KO group.

Without treatment, PT-Atg7-KO and PT-Atg7-WT mice showed similar levels of BUN and serum creatinine, indicative of normal renal function in these mice. At 24 h of LPS treatment, PT-Atg7-KO mice showed significantly higher BUN than PT-Atg7-WT mice (104.74 ± 12.93 vs. 79.80 ± 27.53 mg/dl; Fig. 5c). Serum creatinine in PT-Atg7-KO mice also was also higher than that of wild type (1.02 ± 0.47 vs. 0.77 ± 0.38 mg/dl) (Fig. 5d). Histological analysis also showed that, in comparison with wild type, PT-Atg7-KO mice suffered more severe tissue damage in renal cortex and outer medulla during LPS treatment (Fig. 6a,b). In addition, TUNEL assay showed more apoptotic cells in PT-Atg7-KO kidney tissue (Fig. 6c,d).

## Discussion

In this study, we analyzed the dynamic change of autophagy in LPS-induced endotoxic AKI and further determined the potential pathogenic role of autophagy. Our results provide convincing evidence for the activation of autophagy in kidney tissues following LPS treatment. Notably, renal autophagy was induced rapidly by LPS, followed by the progression from autophagosome to autolysosome, and finally completed by autophagic degradation. Functionally, blockade of autophagy either pharmacologically or genetically exacerbated LPS-induced AKI, suggesting that autophagy plays a protective role in septic/endotoxic AKI.

Autophagy has been well studied in AKI induced by renal ischemia-reperfusion. In nephrotoxic AKI, cisplatin has been shown to activate autophagy in both cultured cells and animals^12^,^13^,^14^,^15^,^16^. On the contrary, very few studies have examined autophagy in septic or endotoxic AKI. Moreover, the results from these few studies are not consistent. In a rat sepsis model of cecal ligation and puncture (CLP), Hsiao and colleagues reported a marked decline of autophagy in kidney proximal tubules^24^. However, Rosengart and colleagues demonstrated autophagy activation at 18 h in LPS-induced endotoxic AKI in mice^25^. The differences between the observations in these studies were most likely caused by the experimental models tested and the time-points monitored. Indeed, the latest work by Karagiannidis *et al.* revealed two peaks of renal autophagy following CLP in rats^26^. While these findings are interesting and sometimes inconsistent, our present study has revealed the dynamics of autophagy in LPS-induced AKI by using *CAG-RFP-EGFP- LC3* autophagy reporter mice, in addition to biochemical analysis of LC3.

The model of *CAG-RFP-EGFP-LC3* autophagy reporter mice was recently established by Hill and colleagues^21^. This model takes advantage of the pH sensitivity of EGFP signal, which is quenched at low or acidic pH. As a result, in autophagosomes with neutral pH, *CAG-RFP-EGFP-LC3* shows both green-GFP and red-RFP fluorescence. However, when autophagy progresses into autolysosomes, pH drops to 4–5, resulting in the quenching of the green-EGFP fluorescence and the appearance of red RFP-only puncta. Using this model, Lin and colleagues recently revealed interesting, dynamic changes of autophagy in kidney tubular cells following renal ischemia-reperfusion injury^20^. It was shown that both RFP and EGFP puncta increased to high levels at 1 day after ischemia, whereas at the end of day 3 the number of EGFP puncta returned to control levels and the high levels of RFP puncta persisted. These impressive findings indicate that autophagy is activated at the early stage of ischemia-reperfusion injury and then progresses to degradation via autolysosome during renal recovery or repair. Using autophagy reporter mice, our present study has now shown the dynamic change of autophagy in LPS- induced endotoxic AKI (Fig. 3). Significant increases in both RFP- and EGFP- LC3 puncta were detected at 8 h of LPS treatment, suggesting an early activation of autophagy in renal tubular cells. At 24 h, RFP-LC3 puncta further increased, but EGFP puncta remained similar to 8 h, suggesting that some autophagosomes had fused with lysosomes to turn into autolysosome. Indeed, the number of autolysosomes was shown to peak at 24 h of LPS treatment (Fig. 3c). By 48 h, the numbers of both RFP- and EGFP- LC3 puncta decreased toward the basal level, indicating the completion of autophagy via degradation. Thus, our results, for the first time, have demonstrated the dynamics of autophagy in renal tubular cells during endotoxic AKI, providing convincing evidence for autophagy activation in this disease condition.

What does autophagy do in LPS-induced kidney injury, and AKI in general? Autophagy was originally described as a cellular response to starvation whereby the cell digests a portion of cytoplasm to produce substrates for ATP generation to maintain cell homeostasis and viability. However, the research in recent years has shown clearly that autophagy is not specific to starvation, but is a general cellular response to stress^8^,^9^,^10^. Autophagy is now known to play crucial roles in development, physiology, and the pathogenesis of a variety of disease^11^. During cell stress, autophagy has been proposed to induce cell death; however, the idea of autophagic cell death has been seriously challenged. In contrast, numerous studies have demonstrated a pro- survival role of autophagy^8^,^9^,^10^,^11^. In kidneys, especially under the condition of ischemic and nephrotoxic AKI, a pro-survival role of autophagy has been demonstrated by the use of pharmacological inhibitors of autophagy as well as autophagy gene knockout models^12^,^13^,^14^,^27^. Nonetheless, our present study is the first to demonstrate a pro-survival or renoprotective role of autophagy in septic/endotoxic AKI by using both pharmacological inhibitors and autophagy gene knockout mouse models. Our results show that chloroquine inhibited renal autophagy during LPS treatment and enhanced kidney injury (Fig. 4). Moreover, proximal tubule-specific Atg7-knockout mice showed more severe AKI flowing LPS treatment (Fig. 6). In these experiments, AKI was indicated by the loss of renal function, tissue damage, and apoptosis. Proximal tubule-specific Atg7-knockout mice showed severe AKI in all these measurements.

While our study demonstrates autophagy activation in endotoxic AKI and further suggests a protective role for autophagy, it remains elusive as to how autophagy is activated under this disease condition. mTOR is well-known to be a negative regulator of autophagy and, in starvation, mTOR suppression leads to autophagy activation. However, mTOR-independent mechanism of autophagy has also been suggested. Very relevant to the present study, Rosengart and colleagues recently showed a surprising observation that autophagy in LPS-induced inflammation and AKI may require mTOR^28^. While this observation is contradictory to the general understanding of negative regulation of autophagy by mTOR, they further suggested that calcium/calmodulin-dependent protein kinase IV may prevent proteosomal degradation of mTOR through GSK-3β to augment autophagy in both the macrophage and the kidney^28^. In support of this possibility, mTOR activation during LPS treatment has been reported. For example, chronic LPS treatment was recently shown to activate mTOR in kidney, probably in macrophages^29^. Apparently, further investigation needs to signaling pathways responsible for the initiation, progression, and completion of autophagy in septic or endotoxic AKI.

## Materials and Methods

### Animals

Atg7^flox/flox^ mice and PEPCK-Cre transgenic mice were originally obtained from Dr. Masaaki Komatsu at Tokyo Metropolitan Institute of Medical Science (Tokyo, Japan)^30^ and Dr. Volker Haase at Vanderbilt University School of Medicine (Nashville, TE)^31^, respectively. C57BL/6 mice were purchased from Jackson Laboratory (Bar Harbor, ME). Kidney proximal tubule-specific Atg7-knockout (PT-Atg7-KO) model was established by breeding Atg7^flox/flox^ mice with PEPCK-Cre transgenic mice as described in our previous work^13^. Briefly, Atg7^flox/flox^ mice were bred to PEPCK-Cre transgenic mice to obtain heterozygous female (Atg7^flox/+^X^cre^X) which was back-crossed with male Atg7^flox/flox^XY mice to generate PT-Atg7-KO mice. The genotypes of PT-Atg7-KO and wild-type (PT-Atg7-WT) mice were verified by PCR-based genotyping. *CAG-RFP-EGFP-LC3* transgenic autophagy reporter mice were originally provided by Dr. Joseph A. Hill at University of Texas Southwestern Medical Center (Dallas, TX)^21^. Male mice at the age of 8–12 weeks were used in this study. All animals were maintained at Charlie Norwood VA Medical Center under 12-hour light/12-hour dark pattern with free access to water and food. All experiments were carried out according to the protocol approved by the Institutional Animal Care and Usage Committee in Charlie Norwood VA Medical Center, Augusta, GA.

### Reagents

Lipopolysaccharide (LPS) from *Escherichia coli* 0111:B4 was purchased from Sigma (catalog no. L2630, St. Louis, MO). The sources of the primary antibodies were as follows: anti-LC3 from Novus Biologicals (Littleton, CO), anti-Atg7, anti-Atg5 and anti-p62 from Abcam (Cambridge, MA), anti- GAPDH from Sigma (St. Louis, MO). All secondary antibodies were purchased from Jackson ImmunoResearch Laboratories Inc (West Grove, PA). Other reagents, including choloquine and paraformaldehyde, were from Sigma (St. Louis, MO).

### LPS-induced AKI

Mice received an intraperitoneal injection of 10 mg/kg body weight LPS, and control mice were injected intraperitoneally (ip) with the equal volume of 0.9% saline. Blood was collected for blood urea nitrogen (BUN) measurement at time 0, 8, 12, 24, and 48 h after LPS injection. Most mice were sacrificed at 24 h after LPS injection, the blood was collected for serum creatinine and BUN measurement, and kidneys were harvested for pathological, immunofluorescence, and immunoblotting analysis.

### Chloroquine treatment

Chloroquine of 60 mg/kg or an equal volume of 0.9% saline was administrated 1 hour before LPS (10 mg/kg) injection and daily afterwards until sacrifice as described in our previous work to inhibit autophagy^12^. The conditions included: normal control group (NC, injection of the equal volume 0.9% saline at corresponding time point), saline + LPS (SL, injection of saline and LPS), chloroquine + LPS (CL, injection of chloroquine and LPS). The protocol of blood collection and kidney harvest were the same as described above.

### Analysis of Renal Function

Blood urea nitrogen (BUN) and serum creatinine were measured to indicate the loss of renal function. Blood samples were collected by cardiac puncture and centrifuged at room temperature to collect the serum. For BUN measurement, the kit from Biotron Diagnostics Inc (Hemet, CA) was used and the reaction was conducted at 100 °C for 12 minutes and then cooled down on ice for 5 minutes. The absorbance of 520 nm was recorded to calculate the value of BUN. For serum creatinine measurement, we used the commercial kit from Stanbio Laboratory (Boerne, TX) and reaction mixture was pre-warmed at 37 °C water bath for 3 minutes, then samples were added to each reaction mixture to record the absorbance of 510 nm at the reaction of 20 seconds and 80 seconds. BUN and serum creatinine levels were calculated based on the standard curves and expressed as mg/dl.

### Renal Histology

For routine analysis, kidney tissues were fixed with 4% paraformaldehyde and embedded in paraffin. Each sample was sectioned at 4μm and stained with standard Hematoxylin-eosin (HE) procedure. A blinded observer was assigned to calculate the percentage of injury in each sample and the renal tubular injury was indicated by tubular dilation/flattening, brush border lost, tubular cast formation, and tubular degeneration and vacuolization.

### TUNNEL Assay of Cell Death

TdT-mediated dUTP mick-end labeling (TUNEL) assay was conducted by using the *in situ* Cell Death Detection kit from Roche Applied Science (Indianapolis, IN) as described in our recent work^32^,^33^. Briefly, paraffin-embedded tissue were deparaffinized with standard protocol and permeabilized with 0.1 M sodium citrate, pH 6.0 at 65 °C for 1 hour. Then the tissue section was incubated with the TUNEL reaction buffer for 1 hour at 37 °C in a humidified chamber. Positive staining was detected by Axioplan2 fluorescence microscope. Each section was selected for 10–20 fields randomly to count positive staining cells.

### Tissue Processing and Examination of Autophagy Reporter Mice

Kidneys were collected from from *CAG-RFP-EGFP-LC3* mice and fixed overnight with 4% paraformaldehyde at 4 °C followed by 30% sucrose immersion for another day. Then the tissue was frozen in OCT (Tissue-Tek, Torrence, CA) compound to cut 4μm cryo-section to store at −80 °C. Frozen sections were washed with 1XPBS for 3 times, 5 minutes for each time, and then mounted to the glass slides with DAPI. LSM780 Upright confocal microscope, ZEN systems and ×630 magnification were used to take pictures. For quantification, 10–20 fields of outer strip of outer medulla were selected randomly and red/green LC3 puncta per proximal tubule were counted.

### Immunoblot analysis

As described previously^15^,^33^,^34^, the whole kidney tissue lysates were extracted with 2% SDS buffer. Protein amounts were determined with BCA reagent from Thermo Scientific. Equal amounts of protein (100 μg for kidney tissue lysates were loaded in each lane of SDS-polyacrylamide electrophoresis gel. Then resolved proteins were transferred to polyvinylidene difluoride membrane, which was then blocked with 5% bovine serum albumin for 1 hour at room temperature and further incubated overnight with a specific primary antibody. The blot membrane was washed with TBST for 4 times and subsequently incubated with horseradish peroxidase-conjugated secondary antibodies. The blot signal was revealed with a chemiluminescence kit (Thermo Scientific).

### Statistical analysis

All values were expressed as mean ± SD. Statistical analysis was conducted by the GraphPad Prism software (San Diego, CA). Comparison between two groups were performed by paired Student t-test or unpaired Student t-test. For multiple comparisons, ANOVA was used. Data were analyzed with a Prism 6.0 software packet (San Diego, CA). Significance was considered by a *P* value of <0.05.

## Additional Information

**How to cite this article**: Mei, S. *et al.* Autophagy is activated to protect against endotoxic acute kidney injury. *Sci. Rep.* **6**, 22171; doi: 10.1038/srep22171 (2016).
